# The association between teacher caring behavior and employment anxiety in female vocational college students: a serial mediation analysis of career decision-making self-efficacy and psychological capital

**DOI:** 10.3389/frcha.2026.1856403

**Published:** 2026-07-14

**Authors:** Juan Liang, Yu Li Liu, Zhanfang Liu

**Affiliations:** 1 Hunan Normal University, Changsha, China; 2 Xiangnan Preschool Education College, Chenzhou, China; 3 Chenzhou Open University, Chenzhou, China; 4 School of Education, Huanggang Normal University, Huanggang, China

**Keywords:** career decision-making self-efficacy, employment anxiety, female vocational college students, psychological capital, teacher caring behavior

## Abstract

**Objective:**

This study aims to examine the association between teachers’ caring behaviors on employment anxiety among female students in vocational colleges, as well as the sequential multiple mediating roles of career decision-making self-efficacy and psychological capital in this association. The findings are intended to offer both theoretical insights and practical guidance for developing psychological interventions to support female students in their transition to employment.

**Methods:**

Based on Social Cognitive Career Theory (SCCT), this study collected data from 1,424 female students enrolled in 10 vocational colleges across Hunan, Jiangxi, and Henan provinces. Participants completed a series of validated instruments, including the Teacher Caring Behavior Questionnaire, the Career Decision-Making Self-Efficacy Scale, the Psychological Capital Questionnaire, and the Employment Anxiety Scale.

**Results:**

The results indicated that: (1) teachers’ caring behaviors were significant negatively correlated with employment anxiety among female vocational college students; (2) career decision-making self-efficacy served as a significant mediator in the association between teacher caring behavior and employment anxiety; (3) psychological capital also functioned as a significant mediator in this association; and (4) career decision-making self-efficacy and psychological capital jointly exerted a significant serial mediating on the link between teacher caring behavior and employment anxiety.

**Conclusion:**

Teacher caring behaviors are negatively correlated with employment anxiety among female vocational college students. In addition, this association can be explained indirectly through a sequential intervening pathway involving career decision-making self-efficacy and psychological capital.

## 1 Introduction

The Outline for Women's Development in China (2021–2030) explicitly emphasizes advancing high-quality vocational education for women, eliminating gender-based employment discrimination, and promoting full employment among female college graduates. According to the 2024 Statistical Monitoring Report on the Implementation of the Outline, released by the National Bureau of Statistics in December 2025, the number of female students enrolled in higher vocational colleges reached 8.656 million in 2024. This represents an increase of 409,000 compared with the previous year, with women accounting for 47.9% of the total student population, an increase of 0.6 percentage points. As the population of female vocational students continues to grow, their employment-related challenges have attracted increasing attention from various sectors of society.

At present, female vocational college students encounter a “dual dilemma” in the labor market. On the one hand, academic discrimination remains widespread. Vocational qualifications are often perceived as less prestigious than undergraduate degrees, with vocational students frequently viewed as possessing comparatively limited competencies (1). As a result, graduates from vocational institutions are more likely to face disadvantages or discriminatory treatment during the job search process (2). One survey reported that 46.7% of vocational students experienced academic discrimination in the employment market (3). On the other hand, structural and societal factors place female workers at a relative disadvantage compared to their male counterparts in terms of wages, occupational status, and opportunities for promotion (4). Empirical studies have shown that female college graduates face less favorable employment outcomes (5), and their levels of employment anxiety are significantly higher than those of male students (6). Employment anxiety among female college students has been conceptualized as comprising three primary dimensions: job-search-related anxiety, career adaptability anxiety, and workplace anxiety (7). As a form of state anxiety, employment anxiety refers to a persistent and intense emotional state characterized by tension and unease, often accompanied by physiological and behavioral responses when individuals confront career-related decisions (8). Although a moderate level of anxiety may enhance motivation during the job search process, excessive employment anxiety can adversely affect both physical and psychological well-being and diminish job-search effectiveness.

According to Social Support Theory, individuals who perceive themselves as being cared for, valued, and respected by others are more likely to have their developmental psychological needs fulfilled, which in turn influences their behavior (9). In the context of employment-related stress, college graduates with lower levels of social support are more susceptible to psychological distress and maladaptive behaviors (10, 11). Research has found that supportive educational environments are associated with alleviated academic and career anxiety, which underscores the importance of teacher-led support systems (12). Beyond the educational context, social connections and supportive environments can mitigate anxiety, particularly for young people undergoing life transitions (13). This aligns with the current study's perspective of viewing supportive relationships as a protective factor against anxiety.

As a key source of social support (14), teachers’ caring behaviors, reflected in academic assistance, emotional support, and behavioral guidance, play a crucial role in shaping students’ mental health (15). Existing studies on teacher caring behavior have predominantly focused on outcomes such as learning anxiety (16), academic performance (17, 18), problem behaviors (19), depression (20), and internet addiction (21). However, limited attention has been given to its correlational relationship with employment anxiety among female vocational college students, particularly regarding the potential intervening mechanisms involved.

## 2 Theoretical basis and research hypotheses

### 2.1 Theoretical basis

Social Cognitive Career Theory (SCCT), which extends Social Cognitive Theory (22) to the vocational domain, highlights the dynamic interplay among background, personal, and behavioral factors in shaping career and educational development. It seeks to explain how individual characteristics and elements of the social environment jointly influence career-related learning processes and decision-making. Centered on core constructs such as self-efficacy, outcome expectations, and personal goals, SCCT proposes a triadic framework of career development that integrates individual cognitive factors (e.g., beliefs and personality traits), environmental influences (e.g., social support and contextual barriers), and behavioral outcomes (23). Building on the foundational “person–environment–behavior” interaction, SCCT further conceptualizes a dynamic cognitive–behavioral pathway specific to career development: “personal attributes/environmental conditions → self-efficacy and outcome expectations → career goals → career behaviors and outcomes” (24, 25). This framework underpins the serial mediation associationl employed in the present study, offering a theoretical lens to elucidate how environmental support correlates with the employment-related emotional adaptation of female vocational college students through the ongoing interaction of cognitive processes and psychological resources.

### 2.2 Research hypotheses

#### 2.2.1 Teacher caring behavior and employment anxiety

Caring is fundamentally characterized by emotional resonance and interpersonal connection, reflecting an individual's sensitivity to others’ emotional states, inner needs, and developmental aspirations (26). Teacher caring behavior refers to a range of actions undertaken by teachers in the processes of instruction, academic guidance, and career mentoring to establish a supportive and nurturing educational environment that addresses students’ psychological needs and interest development (27). It is commonly conceptualized as comprising three dimensions: responsibility, supportiveness, and inclusiveness (28). In this study, teacher caring behavior refers to a series of observable and actionable practices: (1) Academic Responsibility (providing timely feedback, tutoring, and setting high expectations); (2) Emotional Support (active listening, empathy, encouragement, and affirmation); (3) Inclusive Respect (gender equality, non-discrimination, and valuing diverse backgrounds); and (4) Career Guidance (career planning, job-seeking skills training, and provision of relevant information). These behaviors can be manifested in scenarios such as regular teacher-student communication, career counseling sessions, and classroom interactions. According to SCCT, teacher caring behavior, as a key environmental factor, shapes career-related emotions and behaviors by influencing individuals’ cognitive processes and psychological states. A substantial body of empirical research has demonstrated that teacher caring behavior is positively associated with students’ academic achievement (17) and social adaptation (29), while being negatively associated with depression (20). In addition, studies have shown that higher levels of perceived teacher care can enhance students’ sense of belonging and reduce learning-related anxiety (30, 31). Furthermore, teacher support has been found to reduce bullying behaviors and emotional distress among vocational college students (32). For female vocational college students who often face the dual pressures of academic stigma and gender-based disadvantage, teacher caring behavior may serve as a buffering resource, thereby alleviating employment anxiety. Accordingly, we propose Hypothesis H1: Teacher caring behavior is negatively associated with employment anxiety.

#### 2.2.2 The mediating role of career decision-making self-efficacy

Career decision-making self-efficacy is derived from Bandura's theory of self-efficacy, which posits that perceived efficacy influences individuals’ choices of activities and environments, as well as their emotional responses when confronting obstacles. Specifically, career decision-making self-efficacy refers to an individual's self-evaluated confidence in their ability to successfully complete tasks related to career decision-making (33). Within SCCT, it represents a key form of personal self-efficacy that significantly shapes cognitive processes and behavioral outcomes, including career awareness, job-search behaviors, work attitudes, and broader career-related activities (34). Teacher caring behavior can facilitate the development of students’ career cognition by helping them clarify career goals, understand decision-making strategies, and gradually build confidence in their ability to make career-related decisions. Empirical evidence suggests that individuals differ in their responses to the same situations depending on their level of career decision-making self-efficacy (35). Specifically, individuals with high career decision-making self-efficacy are more likely to approach uncertainty in career decision-making with composure and proactivity, and to formulate and implement job-search plans more effectively. In contrast, those with low levels of self-efficacy are more prone to career confusion, self-doubt, and negative emotional responses such as anxiety and avoidance when facing employment pressures. Research further indicates that female students tend to report significantly lower levels of career decision-making self-efficacy than their male counterparts (36), and that career decision-making self-efficacy is significantly negatively associated with employment anxiety (37). Accordingly, we propose Hypothesis H2: Career decision-making self-efficacy is a mediator the association between teacher caring behavior and employment anxiety.

#### 2.2.3 The mediating role of psychological capital

Psychological capital refers to a form of positive psychological resource accumulated during individual growth and development, comprising four key components: optimism, hope, self-efficacy, and resilience (38). In line with the dynamic cognitive–behavioral chain proposed in SCCT, teachers serve as key guides in the academic and career development of vocational college students, and their caring behaviors represent an important environmental influence that fosters positive behavioral patterns and social competencies (15). Empirical research has shown that teacher caring behavior significantly correlates with students’ psychological capital. In particular, teacher behaviors such as encouragement, support, respect, and acceptance can effectively enhance students’ confidence and positive psychological states (39). Within the SCCT framework, psychological capital constitutes an important personal cognitive resource when students make employment-related decisions. In the face of adversity in both academic and career domains, psychological capital plays a vital role in maintaining mental health and enhancing recovery capacity (40). Individuals with higher levels of psychological capital are more likely to maintain a positive outlook under employment pressure, adopt proactive coping strategies, and reduce avoidance behaviors, thereby experiencing lower levels of anxiety. A growing body of research indicates that psychological capital is significantly negatively associated with employment anxiety among college graduates (41–43) and among female college students specifically (44), suggesting that lower levels of psychological capital are associated with higher levels of employment anxiety. Accordingly, we propose Hypothesis H3: Psychological capital is a mediator the association between teacher caring behavior and employment anxiety.

#### 2.2.4 The serial mediating role of career decision-making self-efficacy and psychological capital

Career decision-making self-efficacy and psychological capital are both positive psychological resources, yet they differ fundamentally in conceptual connotation and functional positioning. Career decision-making self-efficacy represents a highly domain-specific cognitive confidence within the employment context, focusing on an individual's judgment of their capability to perform specific tasks such as career choice and job-seeking preparation. In contrast, psychological capital is a cross-situational and relatively stable psychological resource. According to SCCT, environmental support primarily influences domain-specific self-efficacy before gradually generalizing into broader psychological resources. Psychological Capital Theory further posits that successful self-efficacy experiences are a critical prerequisite for the development of psychological capital (38); specifically, increased confidence in specific tasks (e.g., career decision-making) translates into higher levels of optimism, hope, and resilience. Existing empirical studies have supported this positive association (45). Against the backdrop of intensifying employment competition, teacher caring—as a key external support and factor of social persuasion (46)—can bolster female vocational students’ career decision-making self-efficacy, thereby promoting the accumulation of psychological capital. Avey et al. (47) also confirmed that positive self-efficacy experiences can buffer stress and alleviate anxiety. Although previous studies have explored the pathway from psychological capital to career decision-making self-efficacy, this study focuses on how teacher caring, as external environmental support, relates to employment anxiety through the “career decision-making self-efficacy → psychological capital” pathway. This logic aligns closely with the core mechanisms of SCCT and better explains the formation process from external support to long-term psychological adaptation. Based on this, we propose Hypothesis H4: Career decision-making self-efficacy and psychological capital sequentially mediate the association between teacher caring behavior and employment anxiety.

**Figure 1:**
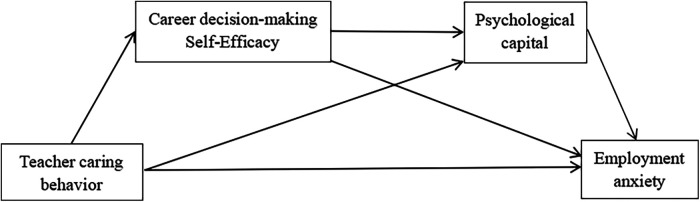
Theoretical model diagram.

Based on the above analysis, the theoretical model is constructed as shown in Figure 1.

## 3 Methods

### 3.1 Participants and procedure

This study was approved by the Ethics Committee of the School of Education at Guangzhou University and conducted in accordance with the Declaration of Helsinki. Written informed consent was obtained from all participants prior to their involvement. Sample size was determined through an *a priori* power analysis and structural equation modeling (SEM) requirements. Using G*Power 3.1 (*α* = 0.05, power = 0.95, f^2^ = 0.15) the required sample size for multiple regression was calculated as *N* = 288. To ensure stability in SEM and mediation association estimates, we aimed to recruit approximately 1,500 participants. Data were collected via an online platform from ten vocational colleges across Hunan, Jiangxi, and Henan provinces. After excluding invalid responses, 1,424 valid questionnaires were retained, resulting in a validity rate of 94.93%.

Regarding demographic background, 150 participants were from urban areas (10.5%), 458 from counties (32.2%), 263 from towns (18.5%), and 553 from rural areas (38.8%). In terms of grade distribution, 914 were graduating seniors (64.2%) and 510 were sophomores (35.8%). Regarding academic specialization, 674 participants were enrolled in liberal arts programs (47.3%), 307 in engineering (21.6%), 38 in arts (2.7%), and 405 in other categories (28.4%). In terms of part-time work experience, 388 participants reported experience related to their major (27.3%), 936 reported experience unrelated to their major (65.7%), and 100 reported no part-time work experience (7.0%). Regarding employment preparation status, 307 participants had secured employment (21.6%), 472 were currently job seeking (33.1%), 297 were preparing for further studies (20.9%), and 348 had not yet developed a clear plan (24.4%).

### 3.2 Measures

#### 3.2.1 Teacher caring behavior scale

The Teacher Caring Behavior Questionnaire developed by Lei (28) was used in this study. The scale consists of 18 items across three dimensions: responsibility, supportiveness, and inclusiveness, with 7, 6, and 5 items in each dimension, respectively. A 5-point Likert scale was adopted (1 = strongly disagree, 5 = strongly agree), with higher scores indicating a greater level of perceived teacher caring behavior. In the present study, the scale demonstrated excellent internal consistency, with a Cronbach's *α* coefficient of 0.98.Confirmatory factor analysis was performed to evaluate the scale's structural validity, and the results revealed an excellent model fit: *χ*^2^/df = 2.87, GFI = 0.99, CFI = 0.99, TLI = 0.99, RMSEA = 0.04, and SRMR = 0.01.

#### 3.2.2 Career decision-making self-efficacy scale

The Career Decision-Making Self-Efficacy Scale, originally developed by Betz et al. (48) and revised by Kuang et al. (49), was employed in this study. The scale comprises 25 items across five dimensions: self-appraisal, information gathering, goal selection, planning, and problem-solving. A 5-point Likert scale was used (1 = no confidence at all, 5 = complete confidence), with higher scores indicating greater career decision-making self-efficacy. In this study, the scale demonstrated excellent internal consistency, with a Cronbach's *α* coefficient of 0.98.Confirmatory factor analysis was conducted to examine the structural validity of the scale. The results demonstrated an acceptable model fit with satisfactory fit indices: *χ*^2^/df = 3.75, GFI = 0.95, CFI = 0.98, TLI = 0.98, RMSEA = 0.04, and SRMR = 0.02.

#### 3.2.3 Psychological capital questionnaire

The Psychological Capital Questionnaire, originally developed by Luthans et al. (68) and revised for Chinese college students by Wang et al. (50), was adopted in this study. The scale comprises 15 items across four dimensions: self-confidence, resilience, optimism, and responsibility. A 5-point Likert scale was used (1 = strongly disagree, 5 = strongly agree), with higher scores indicating a higher level of psychological capital. In this study, the scale demonstrated strong internal consistency, with a Cronbach's *α* coefficient of 0.96.Confirmatory factor analysis was performed to evaluate the scale's structural validity, and the results revealed an excellent model fit: *χ*^2^/df = 4.54, GFI = 0.97, CFI = 0.99, TLI = 0.98, RMSEA = 0.05, and SRMR = 0.02.

#### 3.2.4 Employment anxiety scale

The College Graduate Employment Anxiety Questionnaire, originally developed by Ren (51) and revised by Lin et al. (52), was used in this study. The scale consists of 17 items across two dimensions: physiological/behavioral symptoms and subjective feelings, with Item 2 serving as a validity (lie-detection) item. A 5-point Likert scale was adopted (1 = never, 5 = very often), with higher scores indicating greater levels of employment anxiety. In this study, the scale demonstrated excellent internal consistency, with a Cronbach's *α* coefficient of 0.97.Confirmatory factor analysis was performed to evaluate the scale's structural validity, and the results revealed a good model fit: *χ*^2^/df = 3.56, GFI = 0.99, CFI = 0.99, TLI = 0.99, RMSEA = 0.04, and SRMR = 0.01.

#### 3.2.5 Data analysis

This study employed SPSS 26.0 software to conduct descriptive analyses, group difference tests, and Pearson correlation analyses on the collected data. Model fit was assessed using AMOS. In addition, the PROCESS macro (version 3.5) developed by Hayes was used to examine the mediation associations.

## 4 Results

### 4.1 Common method bias test

Given that all data in this study were collected through self-reports, potential common method bias may exist. Such bias may inflate the direct association between teacher caring behavior and employment anxiety and overestimate the magnitude of the intervening effects of career decision-making self-efficacy and psychological capital. Therefore, Harman's single-factor test was conducted to examine this potential problem. The results identified 11 factors with eigenvalues greater than 1, accounting for 72.3% of the total variance. The first common factor explained 37.0% of the total variance, which was below the critical threshold of 40%. Nevertheless, Harman's single-factor test has limited detection power. To mitigate such risks, multiple procedural controls were implemented in the questionnaire design stage, including anonymous survey participation and the random arrangement of reverse-scored items.

### 4.2 Descriptive statistics and correlation analysis

As shown in Table 1, teacher caring behavior was significantly and negatively correlated with employment anxiety (r = −0.26, *p* < 0.001), indicating a small-to-medium effect size. Despite the statistical significance of this correlation, the relatively weak association suggests that teacher caring exerts a limited independent predictive effect on employment anxiety. In comparison, teacher caring behavior exhibited moderately strong positive correlations with career decision-making self-efficacy (r = 0.50, *p* < 0.001) and psychological capital (r = 0.61, *p* < 0.001). The correlation between career decision-making self-efficacy and psychological capital was even stronger (r = 0.74, *p* < 0.001). Additionally, both career decision-making self-efficacy (r = −0.30, *p* < 0.001) and psychological capital (r = −0.33, *p* < 0.001) demonstrated moderate negative correlations with employment anxiety.

**Table 1:** Descriptive statistics and Pearson correlation analysis.

Variable	M ± SD	1	2	3	4
1. Teacher caring behavior	3.66 ± 0.67				
2. Career decision-making self-efficacy	3.25 ± 0.63	0.50***			
3. Psychological capital	3.43 ± 0.63	0.61***	0.74***		
4. Employment anxiety	2.54 ± 0.99	−0.26***	−0.30***	−0.33***	

*** *p* < 0.001 (the same below).

### 4.3 Test of the serial mediation association

**Figure 2:**
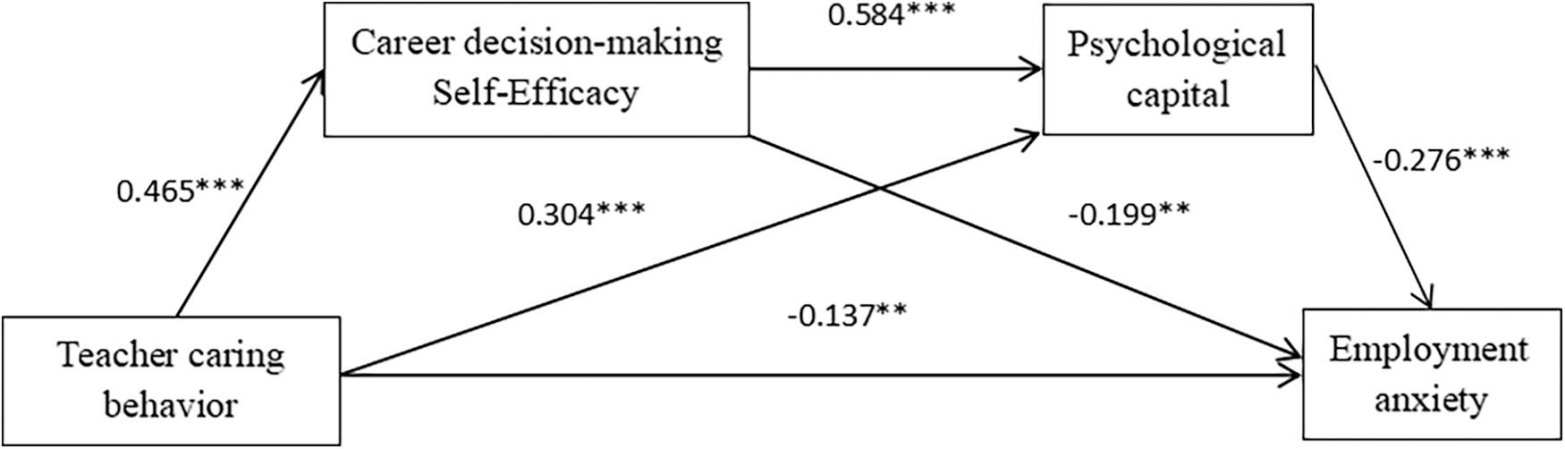
Diagram of the serial mediation model. **p<0.01 and ***p<0.001.

Based on Social Cognitive Career Theory, this study employed structural equation modeling (SEM) to examine the mediating associations of career decision-making self-efficacy and psychological capital in the relationship between teacher caring behavior and employment anxiety among female vocational college students. Model fit was evaluated using widely accepted criteria (53):χ2/df < 5, GFI > 0.90, CFI > 0.90, TLI > 0.90, RMSEA < 0.08, and SRMR < 0.08. The SEM was estimated using maximum likelihood (ML). The model demonstrated a good fit to the data:χ2/df = 3.34, GFI = 0.98, CFI = 0.99, TLI = 0.99, RMSEA = 0.04, SRMR = 0.01, *p* < 0.001. The standardized path coefficients of the serial mediation model are displayed in Figure 2. As shown in Table 2, teacher caring behavior significantly and negatively correlated with employment anxiety (*β* = −0.137, t = −2.641, *p* < 0.01), supporting Hypothesis 1. Teacher caring behavior significantly and positively predicted career decision-making self-efficacy (*β* = 0.465, t = 21.552, *p* < 0.001), and career decision-making self-efficacy significantly and positively predicted psychological capital (*β* = 0.584, t = 31.379, *p* < 0.001). Furthermore, psychological capital significantly and negatively predicted employment anxiety (*β* = −0.276, t = −4.306, *p* < 0.001). In addition, teacher caring behavior significantly and positively predicted psychological capital (*β* = 0.304, t = 17.392, *p* < 0.001), while career decision-making self-efficacy significantly and negatively predicted employment anxiety (*β* = −0.199, t = −3.396, *p* < 0.01).

**Table 2:** Regression analysis of research variables.

Outcome variable	Predictor variable	R^2^	F	*β*	t
Career decision-making self-efficacy	Teacher caring behavior	0.246	464.474	0.465	21.552^***^
Psychological capital	Teacher caring behavior	0.631	1213.009	0.304	17.392^***^
	Career decision-making self-efficacy			0.584	31.379^***^
Employment anxiety	Teacher caring behavior	0.121	64.929	−0.137	−2.641^**^
	Career decision-making self-efficacy			−0.199	−3.396^**^
	Psychological capital			−0.276	−4.306^***^

Bootstrap confidence intervals (percentile method, 5,000 resamples) are reported. β represents the fully standardized effect size.

** *p*<0.01.

*** *p*<0.001.

The Mediation effects were examined using the SPSS PROCESS macro (Model 6), with 5,000 bootstrap resamples to generate 95% bias-corrected confidence intervals. The results indicated that the overall association between teacher caring behavior and employment anxiety was statistically significant (standardized *β* = −0.389, SE = 0.038, 95% CI = [−0.462, −0.315]). The total indirect association of teacher caring behavior with employment anxiety was also significant (*β* = −0.252, SE = 0.038, *p* < 0.001, 95% CI = [−0.326, −0.179]), accounting for 64.78% of the total effect. As shown in Table 3, three distinct indirect pathways were identified. Teacher caring behavior was indirectly associated with reduced employment anxiety through career decision-making self-efficacy (*β* = −0.093, effect size = 23.91%, 95% CI = [−0.157, −0.039], *p* < 0.001) and psychological capital (*β* = −0.084, effect size = 21.59%, 95% CI = [−0.133, −0.039], *p* < 0.001), respectively. Furthermore, a significant sequential mediating pathway was observed, whereby teacher caring behavior correlated with employment anxiety via the chained pathway of career decision-making self-efficacy and psychological capital (*β* = −0.075, effect size = 19.28%, 95% CI = [−0.118, −0.034], *p* < 0.001). The three indirect pathways contributed relatively evenly to the total indirect effect, accounting for 23.91%, 21.59%, and 19.28% respectively, suggesting that no single mediating variable played a dominant role in the underlying mechanism. These findings provide empirical evidence supporting Hypotheses 2, 3, and 4.

**Table 3:** Test of the serial mediation effect.

Item	Path	Effect	Boot SE	Effect Size	BootCI Lower	BootCI Upper
Direct Effect		−0.137	0.047		−0.228	−0.046
Indirect Effect	Teacher caring behavior → Career decision-making self-efficacy → Employment anxiety	−0.093	0.033	23.91%	−0.157	−0.039
	Teacher caring behavior → Psychological capital → Employment anxiety	−0.084	0.024	21.59%	−0.133	−0.039
	Teacher caring behavior → Career decision-making self-efficacy → Psychological capital → Employment anxiety	−0.075	0.021	19.28%	−0.118	−0.034
Total Indirect Effect		−0.252	0.037		−0.326	−0.179
Total Effect		−0.389	0.038		−0.462	−0.315

## 5 Discussion

Social Cognitive Career Theory serves as a valuable framework for exploring career development. Research has consistently demonstrated its utility in predicting career planning, facilitating transitions, and fostering career growth (54–56), as well as in preparing students for college and careers (57, 58). Focusing on female students in Chinese vocational colleges, this study highlights that within China's collectivist culture—where teacher-student relationships carry profound social and emotional significance—teacher care acts as a particularly salient protective factor against academic stigma, gender bias, and employment pressure. This finding echoes the “consistency effect of teacher support” proposed in cross-cultural research, which suggests that teacher support serves as a pivotal predictor of students’ school belonging and academic engagement across both collectivistic and individualistic cultures (59). Nevertheless, in collectivistic cultural contexts, teacher support is more deeply embedded in social structures characterized by high power distance. Beyond providing emotional assistance, teacher caring behavior further undertakes multifaceted functions including social recognition, normative guidance, and relational obligation implication (60).Consequently, our findings further reveal the amplified positive impact of teacher care on female vocational students within this collectivist context. Such cross-cultural nuances not only enrich the international applicability of SCCT but also underscore the necessity of implementing differentiated career psychological interventions for specific demographic groups.

First, teacher caring behavior significantly and negatively correlated with employment anxiety among female vocational college students, thereby supporting H1. From the perspective of person–environment interaction in SCCT, teacher caring behavior serves as a crucial source of social support for female vocational students during the job-search process. It can directly reduce perceived stress and enhance a sense of security, thereby improving emotional adaptation outcomes. Previous research has shown that teacher care behaviors can reduce student anxiety (61).Compared with males, females generally exhibit stronger prosocial tendencies such as enthusiasm, empathy, and compassion (41), which may make them more sensitive to and more likely to internalize caring behaviors from significant others such as teachers. As a result, external environmental support can be more readily transformed into internal psychological security, thereby reshaping negative cognitions and alleviating negative emotions, consistent with previous findings (20). Research has also confirmed that greater perceived care is associated with improved physical and mental health and reduced psychological distress (20). During the job-search process, female vocational college students often face multiple pressures, including academic stigma, gender discrimination, intense employment competition, and limited job opportunities, which increase their susceptibility to self-doubt and anxiety. However, emotional support, inclusive respect, and career guidance provided by teachers can function as an important protective buffer, enabling students to approach employment and career decision-making with greater psychological stability. Notably, although the correlation between teacher caring behavior and students’ employment anxiety reached statistical significance in this study, the effect size was relatively weak. This indicates that teacher caring is not a core dominant predictor of employment anxiety among female vocational college students, and emotional care from teachers alone cannot fundamentally alleviate students’ employment anxiety. Accordingly, university employment education and psychological counseling practices should not overly rely on the single intervention of teacher caring. Instead, teacher emotional and humane support should be integrated into a systematic educational framework that encompasses the improvement of students’ professional literacy, cultivation of employability, provision of employment resources, and empowerment of psychological guidance, so as to construct a comprehensive and multi-dimensional intervention system for mitigating employment anxiety.

Second, career decision-making self-efficacy played a significant mediating role in the relationship between teacher caring behavior and employment anxiety among female vocational college students, thereby supporting H2. Teacher caring behavior not only directly influences students’ employment anxiety but also exerts an indirect effect through career decision-making self-efficacy. Specifically, teacher caring behavior reduces employment anxiety by enhancing students’ confidence in career decision-making. In line with SCCT, self-efficacy is a central determinant of career choice, reflecting individuals’ beliefs in their capability to execute job-search plans and achieve successful employment outcomes (62). For female vocational college students, teachers provide not only emotional support but also strengthen confidence in career decision-making and job-search behaviors through caring practices such as offering employment-related information, job-search skills training, career planning guidance, and decision-making feedback. Higher levels of career decision-making self-efficacy enable students to more actively explore options, make firm choices, and take proactive actions when confronted with uncertainty in the job market, thereby reducing employment anxiety and facilitating smoother employment adaptation. These findings further suggest that enhancing career decision-making self-efficacy is a key leverage point for alleviating employment anxiety among female vocational college students, and that this improvement should be supported through targeted teacher guidance rather than relying solely on individual self-adjustment.

Third, psychological capital played a significant mediating role in the relationship between teacher caring behavior and employment anxiety among female vocational college students, thereby supporting H3. According to SCCT, psychological capital is a set of positive psychological resources developed during the employment process, reflecting individuals’ internal psychological strength and adaptability when facing employment-related stress and challenges. When female vocational college students receive greater emotional support, encouragement, and recognition from teacher caring behaviors, their reservoir of positive psychological resources becomes more enriched. This can effectively offset the depletion of psychological resources under stressful employment conditions, enabling students to cope more effectively with various challenges during the job-search process (63). At the same time, higher levels of psychological capital can stimulate stronger intrinsic motivation, encouraging individuals to integrate self-experiences, clarify career directions, and sustain efforts toward achieving career goals (64), thereby alleviating employment anxiety. Research has consistently demonstrated the positive role of psychological capital in employment competition (65, 66); it enhances job placement success (67), thereby alleviating employment anxiety. Within collectivist cultures, support from authority figures such as teachers exerts a stronger boosting effect on psychological capital, making it easier for individuals to internalize external care into stable, positive psychological resources. For female vocational students facing a “double disadvantage,” psychological capital serves a critical stress-buffering function. Consequently, teacher care indirectly reduces employment anxiety in a more enduring manner by bolstering intrinsic psychological capital.

Fourth, career decision-making self-efficacy and psychological capital exerted a significant serial mediating role on the association between teacher caring behavior and employment anxiety among female vocational college students, which supports Hypothesis 4. Grounded in SCCT, the present findings underscore the dynamic interplay between individual attributes and contextual influences, as well as the synergistic interaction between personal agency and external support (23). A core tenet of SCCT posits that environmental resources shape career-related affective and behavioral outcomes via a sequential pathway of cognitive processes and psychological resources. Consistent with this theoretical framework, teacher caring behavior, as a key form of environmental support, first elevates female vocational students’ career decision-making self-efficacy through emotional support, career guidance, and positive reinforcement. This enhanced self-efficacy subsequently facilitates the development and consolidation of psychological capital. Acting collectively, career decision-making self-efficacy as a cognitive antecedent and psychological capital as an internal psychological resource mediate the transition from external support to adaptive emotional functioning, ultimately mitigating employment anxiety. Notably, the two mediators contributed comparably to the indirect effect, highlighting their balanced importance. Accordingly, interventions should prioritize both constructs equally, adopting integrated strategies to strengthen career decision-making self-efficacy and foster psychological capital concurrently.

## 6 Research contributions and implications

The findings of this study provide meaningful practical guidance for vocational educators, administrators, and career counselors, while offering empirical evidence for the applicability of SCCT in vocational education and among female student populations. First, teacher caring should be regarded as a valuable complementary intervention rather than a sole solution to employment anxiety. Teachers are encouraged to develop a systematic framework of care that emphasizes emotional support, inclusive communication, responsibility-based guidance, and individualized career counseling. Such practices may help alleviate employment anxiety and foster positive psychological resources among female students. Second, vocational institutions may design targeted intervention programs, including career exploration workshops, mock interviews, and career planning courses, to enhance students’ career decision-making self-efficacy and reinforce the pathway linking teacher support to psychological capital. Third, mental health education should integrate the cultivation of psychological capital components—such as resilience, optimism, and hope—to strengthen students’ internal resources for coping with employment-related stress. Finally, addressing the dual challenges of academic stigma and gender discrimination requires career guidance grounded in gender-equality principles, including anti-discrimination education and the promotion of fair employment environments. In summary, this study offers practical implications for vocational colleges to optimize educational practices, improve employment services, and support female students in reducing anxiety and enhancing career confidence and adaptability, thereby contributing to the high-quality development of vocational education and the cultivation of female talent.

## 7 Limitations and future directions

Although this study clarifies underlying mechanism linking teacher caring behavior to employment anxiety among female vocational college students, several limitations should be acknowledged. First, the cross-sectional design limits the ability to draw causal inferences among variables, and the reliance on self-reported data may introduce reporting bias. In addition, potential moderating variables, such as major differences and internship experience, were not fully examined. Future research could adopt longitudinal or tracking designs and integrate multi-source data to enhance the robustness and validity of the findings. Moreover, the research model could be further extended by incorporating additional moderating variables to refine the understanding of the serial mediation mechanism. At the same time, given the specific characteristics of female vocational college students, teacher caring practices and psychological intervention strategies should be further optimized to provide more precise and sustainable employment-related psychological support.

## 8 Conclusion

This study draws the following main conclusions:

1. Teacher caring behavior significantly and negatively correlated with employment anxiety among female vocational college students.
2. Career decision-making self-efficacy served as a significant mediator in the association between teacher caring behavior and employment anxiety among female vocational college students.
3. Psychological capital served as a significant mediator in the association between teacher caring behavior and employment anxiety among female vocational college students.
4. Career decision-making self-efficacy and psychological capital served as a significant serial mediator in the association between teacher caring behavior and employment anxiety among female vocational college students.

## Data Availability

The raw data supporting the conclusions of this article will be made available by the authors, without undue reservation.
